# Neurokinin B Administration Induces Hot Flushes in Women

**DOI:** 10.1038/srep08466

**Published:** 2015-02-16

**Authors:** Channa N. Jayasena, Alexander N. Comninos, Evgenia Stefanopoulou, Adam Buckley, Shakunthala Narayanaswamy, Chioma Izzi-Engbeaya, Ali Abbara, Risheka Ratnasabapathy, Julianne Mogford, Noel Ng, Zubair Sarang, Mohammad A. Ghatei, Stephen R. Bloom, Myra S. Hunter, Waljit S. Dhillo

**Affiliations:** 1 Section of Investigative Medicine, Imperial College London, Hammersmith Hospital, London, UK; 2 Department of Psychology, Institute of Psychiatry, King's College London, Guy's Hospital, London, UK

## Abstract

Neurokinin B (NKB) is a hypothalamic neuropeptide binding preferentially to the neurokinin 3 receptor. Expression of the gene encoding NKB is elevated in postmenopausal women. Furthermore, rodent studies suggest that NKB signalling may mediate menopausal hot flushes. However, the effects of NKB administration on hot flushes have not been investigated in humans. To address this, we performed a randomised, double-blinded, placebo-controlled, 2-way cross-over study. Ten healthy women were admitted to a temperature and humidity-controlled research unit. Participants received 30 minute intravenous infusions of NKB and vehicle in random order. Symptoms, heart rate, blood pressure, sweating and skin temperature were compared between NKB and vehicle in a double-blinded manner. Eight of ten participants experienced flushing during NKB infusion with none experiencing flushing during vehicle infusion (P = 0.0007). Significant elevations in heart rate (P = 0.0106 vs. pre-symptoms), and skin temperature measured using skin probe (P = 0.0258 vs. pre-symptoms) and thermal imaging (P = 0.0491 vs. pre-symptoms) characteristic of menopausal flushing were observed during hot flush episodes. Our findings provide evidence that NKB administration can cause hot flushes in women. Further studies are required to determine if pharmacological blockade of NKB signalling could inhibit hot flushes during the menopause and during treatment for sex-steroid dependent cancers.

Hot flushes (or flashes) are intermittent episodes of sweating and heat sensation associated with decreasing circulating sex steroid levels. Hot flushes are experienced by 70–80% of women during the menopausal transition and postmenopause, and can have a significant negative impact on quality of life^1^,^2^,^3^. Hot flushes are also experienced by many patients undergoing sex steroid deprivation therapy for breast and prostate cancer^4^. Hormone replacement therapy (HRT) is the most effective and commonly used pharmacological treatment for menopausal hot flushes. However, current guidelines recommend a limited duration of HRT therapy due to the associated risks (e.g. breast cancer, coronary artery disease, stroke, and thromboembolism)^5^. Furthermore HRT is contra-indicated in many patients (e.g. history of thromboembolic disease, breast/endometrial cancer, liver disease), and may be unsuitable for patients with hormone-dependent cancers undergoing sex steroid deprivation therapy^5^. Other therapies such as selective serotonin reuptake inhibitors (SSRIs), gabapentin, and clonidine can be used to treat hot flushes. However these treatments are less effective than HRT and have a number of commonly associated adverse effects such as sedation, nausea and orthostatic hypotension^2^,^6^,^7^. There is therefore a major need to identify therapeutic targets to treat hot flushes more effectively.

Menopausal flushes are triggered by a fall in circulating sex steroid levels. Furthermore, thermoregulatory centres in the hypothalamus are thought to play a crucial role in mediating the hot flush response. However, the central mechanisms through which sex steroid deficiency triggers flushing remain unclear. Learning more about the mechanisms governing hot flushes may allow us to identify novel therapeutic targets to better treat patients affected by these symptoms. It has recently emerged that the hypothalamic hormone, neurokinin B (NKB) may play an important role in linking oestrogen deficiency to hot flushes. NKB is a decapeptide and a member of the tachykinin family of peptides^8^. In humans, NKB is encoded by the TAC3 gene and binds preferentially to the neurokinin 3 receptor (NK3R, encoded by the TAC3R gene)^9^. Hypothalamic NKB expression is elevated during menopause, and restored to normal levels with oestrogen replacement in monkeys^10^,^11^,^12^. Furthermore, rodent studies suggest that the flushing response to oestrogen deficiency may be dependent on endogenous hypothalamic NKB signalling^13^. These animal data combined suggest that NKB signalling may be crucial in the hot flush aetiology.

The question of whether exogenous NKB can induce hot flushes in humans has not yet been investigated. We performed a randomised, double-blinded, placebo-controlled, two-way cross-over study to determine the effects of NKB administration on hot flushes in women.

## Methods

All experimental protocols were approved by the National Research Ethics Service (NRES) Committee London – West London (registration number 10/H0707/68) and were performed in accordance with the Declaration of Helsinki. Written informed consent was obtained from all participants. See Supplementary Methods online for participant characteristics, peptide characteristics, and protocol for blood collection and analysis.

### Study 1: Symptom assessment study during intravenous (iv) infusion of NKB to healthy women

**Figure 1:**
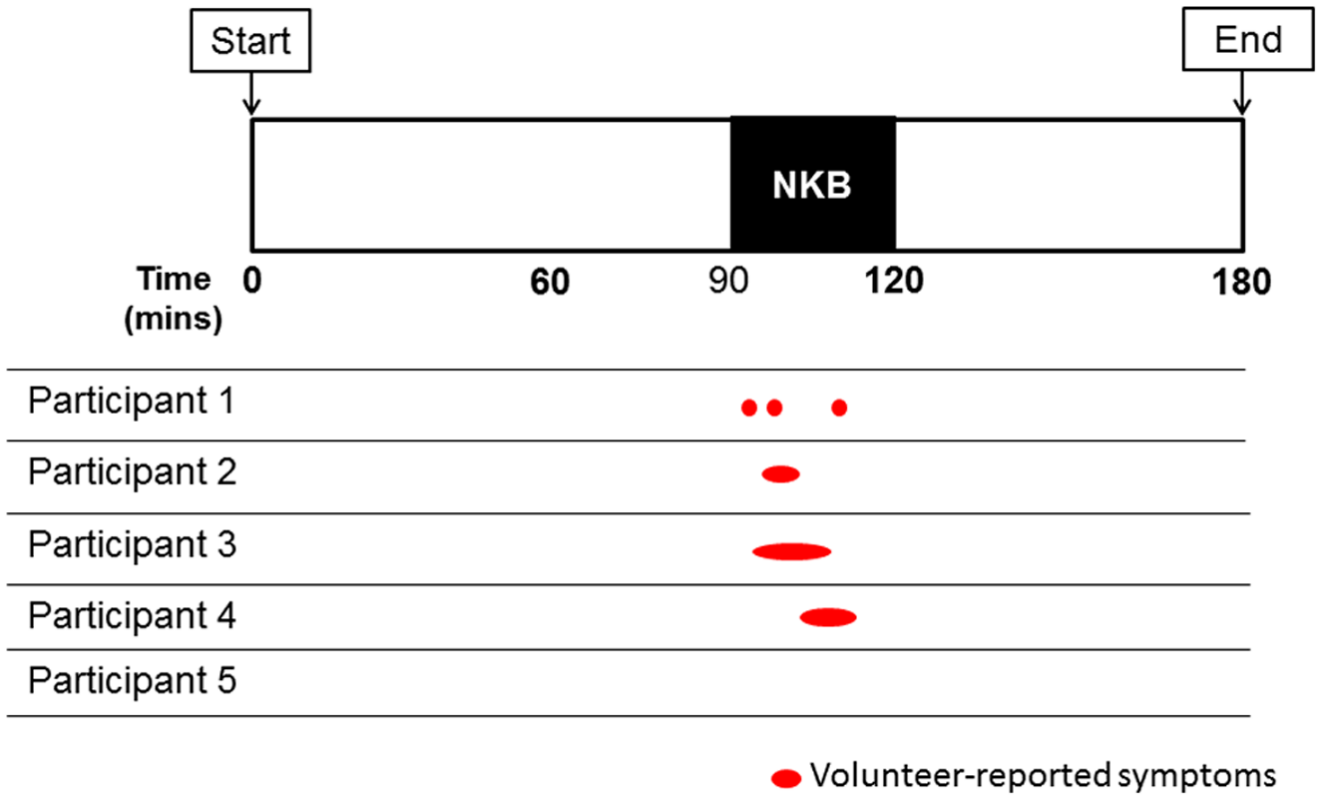
Participant-reported hot flush symptoms during 30 minute intravenous infusion of neurokinin B (Study 1). Five healthy women were monitored for 180 minutes in a Clinical Research Unit. They were administered an intravenous infusion of neurokinin B (NKB, 5.12 nmol/kg/h) between 90–120 minutes. Red ovals represent timings and duration of participant-reported hot flush symptoms. Hot flush symptoms were reported by four out of five participants.

Participants were admitted in the morning to our Clinical Investigation Unit between days 3 and 10 of their menstrual cycle, and asked to lay supine for the 180 minute study duration (Figure 1). For participant characteristics see Supplementary Table S1 online. A cannula was inserted into a large forearm vein and NKB (5.12 nmol/kg/min) was administered by intravenous (iv) infusion for 30 minutes commencing at t = 90 minutes. We selected this dose of NKB as it was the maximal dose that was well-tolerated dose in our previous studies^14^. NKB was dissolved in saline containing gelofusin (5% vol/vol) (B.Braun Medical, Sheffield, UK) to minimize peptide adsorption^15^. Participants (but not investigators) were blinded as to the identity of the infusion (NKB or vehicle, however all participants received NKB). Participants were informed before the study that they may experience body sensations or symptoms and they were asked to report these in real time during the study. Participants were not specifically informed that they may experience ‘hot flush’ symptoms so as to minimise reporting bias. We employed subjective reporting of ‘hot flush’ symptoms as it is currently the most accurate method for detection and assessment of hot flushes (see review by Sievert) and is therefore the preferred method for clinical studies in the field^16^,^17^,^18^,^19^.

### Study 2: Detailed physiological assessment study during iv infusion of NKB and vehicle to healthy women

Having observed in Study 1 that participants reported flushing during NKB infusion we carried out a double-blinded placebo-controlled study. This was performed in a climate-controlled Clinical Research Unit with ambient temperature 24°C and humidity 50% as established in previous studies to be a suitable environment to investigate menopausal flushing^20^,^21^,^22^,^23^. Participants were admitted on a morning between days 3 and 10 of their menstrual cycle, and asked to lay supine for the 270 minute study duration (Supplementary Figure S1 online). For participant characteristics see Supplementary Table S2 online. A cannula was inserted into a large forearm vein in both arms: one for collection of blood, and the second for vehicle (gelofusin) or NKB infusion. 30 minute iv infusion of either NKB (5.12 nmol/kg/h) or vehicle (equivalent volume) were commenced at t = 60 minutes and t = 180minutes (90 minute washout period between infusions), by an investigator blinded to the identity of each infusion. Each participant received one NKB infusion and one vehicle infusion, randomised and prepared by an independent investigator. Participants were informed that they could receive any of the following: NKB followed by vehicle; vehicle followed by NKB; NKB twice; vehicle twice. This strategy ensured integrity of blinding, since any symptoms experienced during the first infusion would not allow participants to automatically deduce the identity of the second infusion.

In order to prevent confounding factors which may affect flushing, participants were asked to refrain from hot drinks, caffeine, alcohol and spicy foods for 12 hours preceding the study start and for the duration of the study. All participants wore light cotton standard hospital gowns. All studies commenced between 9.30–10.30am. Two experienced physicians were in attendance at all times. As in Study 1, subjective hot flush symptoms were self-reported by the participants. In addition heart rate, blood pressure, sweating and skin temperature monitoring were performed as detailed below:

### Heart rate (HR) and mean arterial blood pressure (MAP)

HR was recorded minutely for 20 minutes pre-infusion until 20 minutes post-infusion. MAP was recorded at 5 minute intervals (to minimize discomfort) for 20 minutes pre-infusion until 20 minutes post-infusion. At other times HR and MAP were recorded at 10 minute intervals (Supplementary Figure S1 online). MAP (in mmHg) was calculated using the following standard formula: MAP = ((2 x diastolic BP) + systolic BP)/3.

### Skin temperature

Skin temperature was recorded each minute for 20 minutes pre-infusion until 20 minutes post-infusion and at 10 minute intervals at other times. Skin temperature was measured by skin temperature probe attached to the neck (Mindray, Huntingdon, UK) and by thermal imaging camera (T440Bx, Flir, Wilsonville, USA). Thermal imaging temperature values were determined by in-built software which recorded the highest temperature point in a box constructed to contain the shoulders and head of the participant.

### Skin conductance

Increased skin conductance is an objective marker of sweating which occurs in menopausal flushing. Sternal skin conductance (SSC) was measured using a previously described Bahr hot flush monitor that measured SSC in microsiemens (μS) every 10 seconds by passing an electric current across two electrodes attached to the sternal region of the chest (Simplex Scientific, Wisconsin, USA)^19^,^24^,^25^.

### Symptoms

On arrival on the study day participants were asked to report their general stress level that day on a scale 1–3 (1-no stress, 2-mild stress, 3-high stress) (Table 1). They were also asked to verbally report any symptoms of any nature that they experienced including their frequency and severity to the study investigators in real-time during the study. As in Study 1, participants were informed before the study that they may experience body sensations or symptoms but were not specifically informed that they may experience ‘hot flush’ symptoms so as to minimise reporting bias. Symptom data was collected by an independent investigator blinded to the identities of infusions administered during the study.

### Reproductive hormones

Blood was collected for luteinizing hormone (LH), follicle stimulating hormone (FSH) and estradiol (E2) at intervals of 10 minutes throughout the study (see Supplementary Methods online).

### Data analysis

The occurrence of flushing was analysed by two-tailed Fisher's exact test. Physiological data passed Kolmogorov-Smirnov testing for normality. Paired means were compared using two-tailed paired t-tests and change in means were compared using two-tailed one-sample t-tests (equivalent results). P < 0.05 was considered statistically significant. Data are presented as mean ± standard error of mean (SEM).

## Results

### Study 1: Effects of 30 minute intravenous infusion of NKB on flushing symptoms in healthy women

We performed a pilot study investigating whether a 30 minute infusion of 5.12 nmol/kg/h NKB could induce flushing symptoms in healthy women during the follicular phase of menstrual cycle.

Flushing symptoms (e.g. warmth, heat, sweating) were only reported during NKB infusion with no flushing symptoms reported during the 90 minutes pre-NKB infusion or the 60 minutes post-NKB infusion. Flushing was reported by four out of the five participants during NKB infusion (Figure 1). Three of the four participants experiencing flushing reported a single flushing episode during NKB infusion, and one participant experienced three flushing episodes during NKB infusion.

### Study 2: Effects of 30 minute intravenous infusion of NKB versus vehicle on symptoms and objective clinical markers of flushing in healthy women

To determine the effects of NKB on flushing in further detail, ten healthy women were administered a 30 minute intravenous infusion of vehicle and a 30 minute intravenous infusion of 5.12 nmol/kg/h NKB during the same study visit, in random order (Supplementary Figure S1 online).

### Flushing episodes during vehicle and NKB

**Figure 2:**
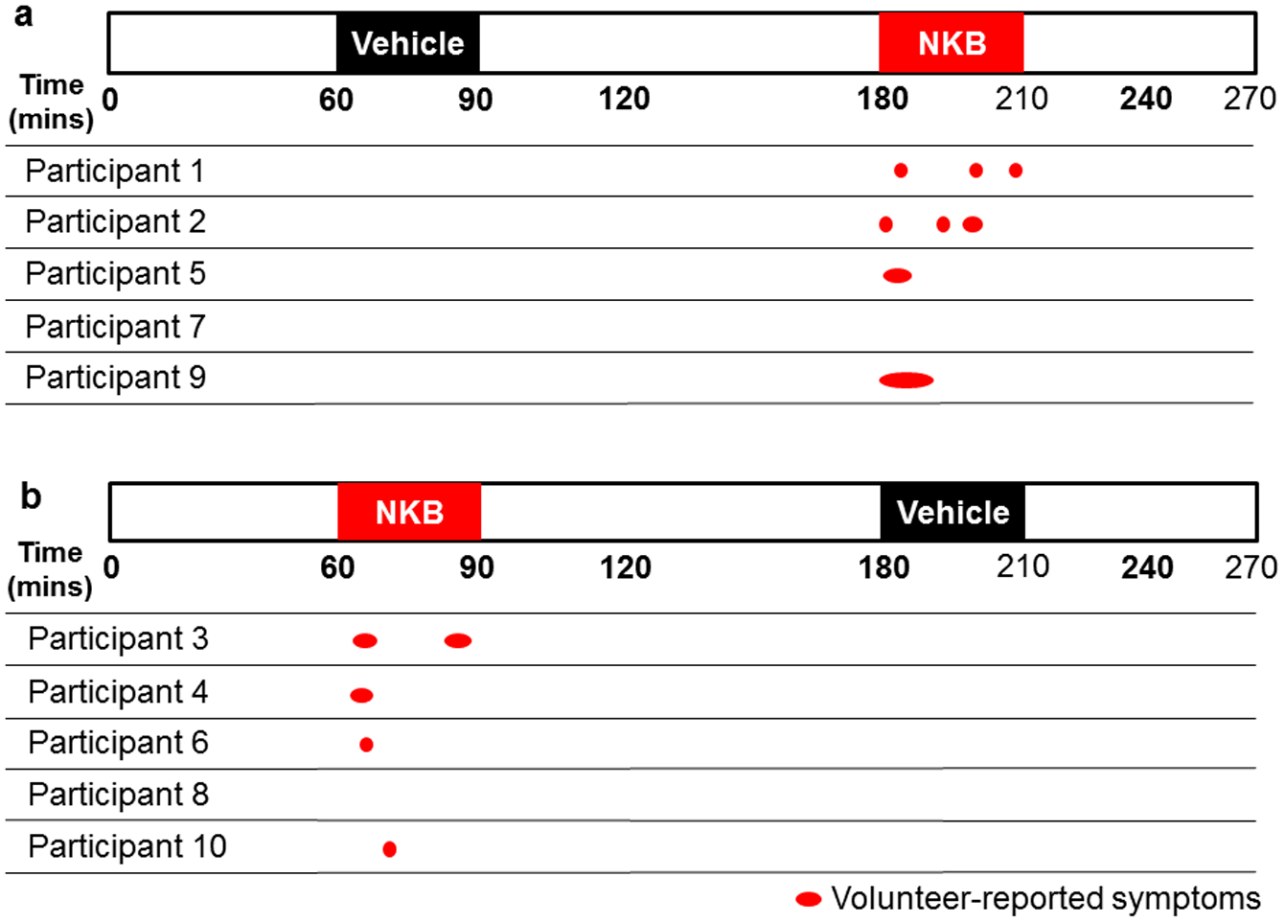
Participant-reported hot flush symptoms during double-blinded administration of vehicle and neurokinin B (Study 2). The order of infusions was randomised by an independent investigator. (a) Participants 1,2,5,7 and 9 received vehicle infusion first and NKB infusion second. (b) Participants 3,4,6,8 and 10 received NKB infusion first then vehicle infusion second. Red ovals represent timings and duration of participant-reported hot flush symptoms. Hot flush symptoms were reported by eight out of ten participants. There were a total of thirteen separate flushing episodes.

No flushing symptoms were recorded during infusion of vehicle in healthy women (Figure 2). Flushing symptoms were only reported during NKB infusion and ranged from mild to strong (mean severity 2/3 = strong heat sensation, See Table 1). During NKB infusion eight out of ten participants experienced flushing (P = 0.007 vs. vehicle) (of these participants, five experienced a single flushing episode, one experienced two flushing episodes, and two participants each experienced three flushing episodes, Figure 2 and Table 1). All first flushing episodes commenced between 1–12 minutes after NKB infusion initiation (Figure 2). As high stress levels are known to influence hot flush reporting in menopausal women, we collected pre-study stress scores^2^,^3^. No participant reported high pre-study stress levels (See Table 1).

Concordant increases in sternal skin conductance (suggestive of sweating) were evident in six of the eight participants at the time of flushing during their NKB infusion (concordance was defined as an objectively-measured sternal skin conductance response corroborated by a subjective self-report of a flush^19^).

### Changes in heart rate, temperature and blood pressure during flushing episodes

**Figure 3:**
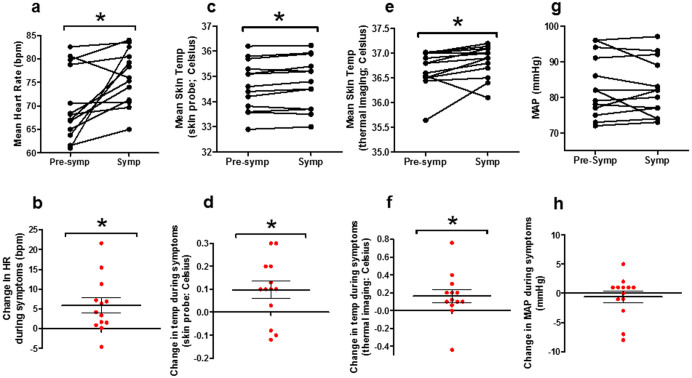
Physiological changes associated with hot flush episodes in healthy women. Mean heart rate (a), skin temperature by skin probe (c), skin temperature by thermal imaging (e) and mean arterial pressure (MAP) (g), during 5 minute period pre-symptom onset (Pre-symp) and during symptom period (Symp) using the minutely recordings. Parts b, d, f and h represent change in respective physiological parameter when compared with pre-symptom level. As MAP was recorded every 5 minutes (rather than minutely so as to avoid discomfort), the MAP measurement immediately prior to symptoms was compared to that at symptom onset or closest after symptom onset. Data presented as mean ± SEM. *P < 0.05.

We investigated specifically whether HR, temperature (measured using skin temperature probe and thermal imaging camera), or MAP were altered specifically during flushing symptoms occurring during NKB infusion. Mean HR increased significantly during flushing symptoms when compared with the pre-symptom period (mean HR in bpm: 70.3 ± 2.1, pre-symptoms; 76.2 ± 1.6, symptoms, P = 0.0106 vs. pre-symptoms) (Figure 3a–b). Mean skin temperature increased significantly during flushing symptoms when compared with the pre-symptom period, whether measured using skin probe (mean temperature in Celsius: 34.6 ± 0.2, pre-symptoms; 34.7 ±0 .3, symptoms, P = 0.0258 vs. pre-symptoms) or using thermal imaging camera (mean temperature in Celsius: 36.7 ± 0.1, pre-symptoms; 36.8 ± 0.1, symptoms, P = 0.0491 vs. pre-symptoms) (Figure 3c–f). No significant change in MAP was observed flushing symptoms when compared with the pre-symptom period (mean MAP in mmHg: 83.2 ± 8.6, pre-symptoms; 82.5 ± 2.2, symptoms, P = 0.546 vs. pre-symptoms) (Figure 3g–h).

### Overall changes in heart rate, temperature and blood pressure during entire vehicle and NKB infusions

No overall changes in HR, skin temperature (measured using skin probe or thermal imaging camera), or mean arterial blood pressure (MAP) were observed between vehicle and NKB infusions when comparing the measurements over the entire duration of the infusion (Supplementary Figure S2 online); hence physiological changes were specific to hot flush episodes and not present for the duration of NKB infusion.

### Changes in serum reproductive hormones during vehicle and NKB

**Figure 4:**
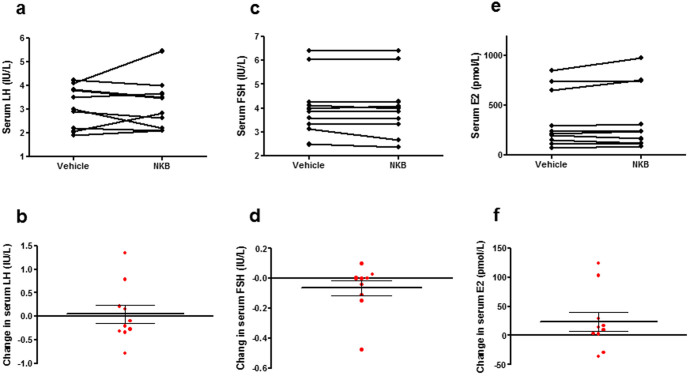
Reproductive hormone changes during neurokinin B and vehicle infusion. Serum luteinizing hormone (LH) (a), serum follicle stimulating hormone (FSH) (c) and serum estradiol (e) levels during neurokinin B (NKB) and vehicle infusion. Parts b, d and f represent change in respective parameter when compared with vehicle. Data presented as mean ± SEM.

No significant changes in serum LH, FSH or estradiol were observed during the entire 30 minute NKB infusion period when compared with vehicle infusion period (Figure 4).

## Discussion

Millions of post-menopausal women and patients undergoing cancer treatments suffer hot flushes worldwide^26^. An improved understanding of the central mechanisms responsible for hot flushes may lead to novel therapies for affected patients, particularly those in whom HRT is contraindicated. Recent data have implicated NKB signalling as an important mediator in menopausal flushing. Hypothalamic NKB neurones exhibit marked hypertrophy and NKB expression increases following the human menopause^27^. Moreover, hypothalamic NKB neurones also undergo hypertrophy and NKB expression increases following ovariectomy in monkeys, while estradiol replacement restores NKB neurones to a size comparable to ovary-intact monkeys^10^,^12^. Furthermore, ablating NKB receptor-expressing neurones (NK3R) within the hypothalamic arcuate nucleus using NK3R-saporin, blocks the typical changes in tail skin and core body temperature in observed in rats following ovariectomy^13^. The effects of NKB on flushing may be mediated within the hypothalamic median preoptic nucleus (MnPO). The MnPO expresses the neurokinin 3 receptor (NK3R), and receives information from warm-sensitive, cutaneous thermoreceptors and projects to CNS centres to modulate heat dissipation effectors^28^,^29^. C-fos expression (a marker of neuronal activation) in the MnPO increases in ovariectomised rats when compared with ovariectomised and estrogen-replaced rats^30^. Furthermore, micro-infusion of the NK3R agonist, senktide, into the MnPO reduces core temperature^30^.

Collectively, the current and previous studies suggest that an increase in NKB signalling may trigger hot flushes during oestrogen sex steroid deficiency. Our data suggests that intravenous infusion of NKB induces hot flushes in healthy women. This is consistent with previous rodent data implicating the NKB signalling pathway as a novel therapeutic target for menopausal hot flushes. A number of NK3R antagonists have already been evaluated in humans for the potential treatment of schizophrenia^31^. It would therefore be interesting in future studies to test whether NK3R antagonists could inhibit endogenous hot flushes in patients with sex steroid deficiency.

We took a number of precautions during the current study to optimise data quality and reduce bias. We used subjective reports of flushing as the primary endpoint of this study, since it is the gold-standard diagnostic measure (and the definition, itself) of hot flushes. We also measured objective clinical indices such as skin temperature, HR and skin conductance which are commonly altered during natural menopausal flushing episodes^32^,^33^,^34^,^35^,^36^,^37^. All study visits were scheduled during days 3 to 10 of the menstrual cycle (follicular phase) when core body temperature is most stable. In addition participants received standardised instructions from a study investigator prior to each visit, and were asked to verbally report frequency and severity of any body sensations or symptoms in real time during the study. Furthermore participants were not specifically informed that they may experience ‘hot flush’ symptoms to avoid reporting bias. During Study 2, participants received one infusion of vehicle and one infusion of NKB, in random order; therefore in order to prevent the identity of the second infusion being deduced from symptoms experienced during the first infusion, all participants and bedside investigators were intentionally told that either or both infusions could contain NKB (when in fact, only one of the two infusions would contain NKB). Finally, Study 2 allowed us to compare the effects of vehicle and NKB on flushing during the same study visit. Our data showed that the primary endpoint, i.e. hot flush symptom, was only observed during NKB infusion in healthy premenopausal women.

Menopausal flushing is known to be associated with increases in heart rate and skin temperature^2^. During menopausal hot flushes, HR has been reported to increase by approximately 5–15 beats per minute without coincident change in blood pressure^35^, and increases in skin temperature of 0.2–1.0°C have also been reported^32^,^38^. Indeed, during the current study, both HR and skin temperature increased significantly and by similar magnitudes to natural menopausal flushes. In addition, it is important to note that overall mean levels of HR, MAP and skin temperature were similar between *entire* NKB and vehicle infusions in healthy women, which suggests that NKB *per se* does not affect these parameters (i.e. significant changes were specific and confined to hot flush episodes rather than over the *entire* infusion duration). Our results therefore suggest that NKB elicits hot flushing episodes in women, which are accompanied by objective physiological effects similar to those observed in menopausal flushes. Sternal skin conductance, a surrogate marker of sweating also showed increases in 6/8 women in study 2, at the time of flushing episodes reported during NKB administration.

NKB is co-expressed within the arcuate nucleus with two other neuropeptides (kisspeptin and dynorphin) in kisspeptin-dynorphin-neurokinin B (KNDy) neurones, which regulate the secretion of gonadotrophin releasing hormone^39^. Inactivating mutations in TAC3 or TAC3R cause pubertal failure in humans^40^,^41^. It was therefore important to measure any effects of NKB on reproductive hormone release. In this study we found at the dose tested, NKB had no significant effects on reproductive hormone release (consistent with our previous study investigating the effects of intravenous NKB administration on reproductive hormone secretion in healthy men and women^14^).

It is interesting to consider whether the effects of NKB we have observed in this study are due to NKB action at central or peripheral NK3Rs. The various physiological parameters were not raised throughout the NKB infusion period, only during the acute intermittent hot flushes which favours a central site of NKB action. This is in keeping with data from animals which shows that microinfusion of NK3 agonist (senktide) centrally into the rat MnPO modulates body temperature^30^. Furthermore, ablation of NK3-expressing neurones inhibits cutaneous vasodilation in rats as measured by tail-skin temperature^13^. However, NKB administration was not associated with gonadotropin secretion and hence this may suggest NKB actions at peripheral NK3Rs also contribute to the effects observed in our study. Whilst TAC3 and TAC3R genes are predominantly expressed centrally there is also expression in several peripheral tissues including the reproductive, gastrointestinal and respiratory tracts with putative contributory roles in epithelial cell function, gastric motility, and airway physiology amongst others^42^. Hence although the effects of NKB observed in the current study are consistent with a central site of NKB action, we cannot exclude the possibility that NKB acting at peripheral NK3Rs could be contributing to the effects we have observed.

In summary, our data shows that intravenous infusion of NKB acutely induces hot flushes in premenopausal healthy women. Further studies are required to determine if pharmacological blockade of NKB signalling could inhibit hot flushes associated with the menopause and treatment for sex-steroid dependent cancers.

## Author Contributions

C.J., A.C., E.S., M.H. and W.D. designed the study. A.C., A.B., S.N., C.I., A.A., R.R., J.M., N.N. and Z.S. performed the studies. M.G. and S.B. provided technical expertise and reagents for assays. C.J., A.C., E.S. and A.B. analysed the data. C.J., A.C. and W.D. wrote the manuscript. All authors reviewed the manuscript.

## Supplementary Material

Supplementary Information Supplementary Information

## Figures and Tables

**Table 1:** Participant-reported symptoms during double-blinded administration of vehicle and neurokinin B (Study 2). Ten healthy women were administered 30 min intravenous infusions of vehicle and neurokinin B (NKB, 5.12 nmol/kg/h) with a 90 min interval between infusions. Vehicle was administered first in participants 1,2,5,7 and 9, and NKB was administered first in participants 3,4,6,8 and 10. Participants were asked to verbally report any symptoms including their frequency and severity (0 no symptom, 1 mild heat sensation, 2 strong heat sensation and able to continue with general activity, 3 strong heat sensation unable to continue with current activity) to the study investigators in real-time during the study. Pre-study stress scores (1 low, 2 medium, 3 high) were recorded on arrival for the study

Participant Number	Pre-Study Stress Score (1–3)	Participant-reported symptoms		Maximum symptoms severity (0–3)
		1^st^ infusion	2^nd^ infusion	
		**VEHICLE**	**NKB**	
1	1	None	Hot forehead, Hot ears, Headache, Warm	2
2	1	None	Hot face, Facial flushing, Headache, Warm	3
5	2	None	Hot face, ‘Hot air blowing on face’, Headache, Warm	2
7	1	None	None	0
9	1	None	Flushing, Clammy, ‘Face burning’, Warm face/arms	2
		**NKB**	**VEHICLE**	
3	2	Cheeks flushing, Warm	None	2
4	1	Warm face, Cold following warm	None	2
8	1	None	None	0
6	1	Hot head, Sweating, Head tingling, Clammy, Warm	None	1
10	1	Feels hot, Face sweating, Warm	None	1

## References

[b1] Carpenter J. S., Johnson D., Wagner L. & Andrykowski M. Hot flashes and related outcomes in breast cancer survivors and matched comparison women. Oncol. Nurs. Forum 29, E16–25, 10.1188/02.ONF.E16-E25 (2002). 11979290

[b2] Archer D. F. *et al.* Menopausal hot flushes and night sweats: where are we now? Climacteric 14, 515–528, 10.3109/13697137.2011.608596 (2011). 21848495

[b3] Ayers B. & Hunter M. S. Health-related quality of life of women with menopausal hot flushes and night sweats. Climacteric 16, 235–239, 10.3109/13697137.2012.688078 (2013). 22809134

[b4] Adelson K. B., Loprinzi C. L. & Hershman D. L. Treatment of hot flushes in breast and prostate cancer. Expert Opin. Pharmacother. 6, 1095–1106, 10.1517/14656566.6.7.1095 (2005). 15957964

[b5] de Villiers T. J. *et al.* Global Consensus Statement on menopausal hormone therapy. Maturitas 74, 391–392, 10.1016/j.maturitas.2013.02.001 (2013). 23497918

[b6] Rada G. *et al.* Non-hormonal interventions for hot flushes in women with a history of breast cancer. Cochrane Database Syst Rev, CD004923, 10.1002/14651858.CD004923.pub2 (2010). 20824841 PMC13500181

[b7] Frisk J. Managing hot flushes in men after prostate cancer--a systematic review. Maturitas 65, 15–22, 10.1016/j.maturitas.2009.10.017 (2010). 19962840

[b8] Maggio J. E. Tachykinins. Annu. Rev. Neurosci. 11, 13–28, 10.1146/annurev.ne.11.030188.000305 (1988). 3284438

[b9] Page N. M. New challenges in the study of the mammalian tachykinins. Peptides 26, 1356–1368, 10.1016/j.peptides.2005.03.030 (2005). 16042976

[b10] Rometo A. M., Krajewski S. J., Voytko M. L. & Rance N. E. Hypertrophy and increased kisspeptin gene expression in the hypothalamic infundibular nucleus of postmenopausal women and ovariectomized monkeys. J. Clin. Endocrinol. Metab. 92, 2744–2750, 10.1210/jc.2007-0553 (2007). 17488799

[b11] Sandoval-Guzman T. & Rance N. E. Central injection of senktide, an NK3 receptor agonist, or neuropeptide Y inhibits LH secretion and induces different patterns of Fos expression in the rat hypothalamus. Brain Res. 1026, 307–312, 10.1016/j.brainres.2004.08.026 (2004). 15488494

[b12] Abel T. W., Voytko M. L. & Rance N. E. The effects of hormone replacement therapy on hypothalamic neuropeptide gene expression in a primate model of menopause. J. Clin. Endocrinol. Metab. 84, 2111–2118, 10.1210/jcem.84.6.5689 (1999). 10372719

[b13] Mittelman-Smith M. A., Williams H., Krajewski-Hall S. J., McMullen N. T. & Rance N. E. Role for kisspeptin/neurokinin B/dynorphin (KNDy) neurons in cutaneous vasodilatation and the estrogen modulation of body temperature. Proc. Natl. Acad. Sci. U. S. A. 109, 19846–19851, 10.1073/pnas.1211517109 (2012). 23150555 PMC3511761

[b14] Jayasena C. N. *et al.* Effects of neurokinin B administration on reproductive hormone secretion in healthy men and women. J. Clin. Endocrinol. Metab. 99, E19–27, 10.1210/jc.2012-2880 (2014). 24170109 PMC3952021

[b15] Kraegen E. W., Lazarus L., Meler H., Campbell L. & Chia Y. O. Carrier solutions for low-level intravenous insulin infusion. Br. Med. J. 3, 464–466 (1975). 1156820 10.1136/bmj.3.5981.464 PMC1674251

[b16] Sievert L. L. Subjective and objective measures of hot flashes. Am. J. Hum. Biol. 25, 573-580, 10.1002/ajhb.22415 (2013). 23897855

[b17] Joffe H. *et al.* Low-dose estradiol and the serotonin-norepinephrine reuptake inhibitor venlafaxine for vasomotor symptoms: a randomized clinical trial. JAMA internal medicine 174, 1058–1066, 10.1001/jamainternmed.2014.1891 (2014). 24861828 PMC4179877

[b18] Freeman E. W. *et al.* Efficacy of escitalopram for hot flashes in healthy menopausal women: a randomized controlled trial. JAMA 305, 267–274, 10.1001/jama.2010.2016 (2011). 21245182 PMC3129746

[b19] Mann E. & Hunter M. S. Concordance between self-reported and sternal skin conductance measures of hot flushes in symptomatic perimenopausal and postmenopausal women: a systematic review. Menopause 18, 709–722, 10.1097/gme.0b013e318204a1fb (2011). 21326119

[b20] de Bakker I. P. & Everaerd W. Measurement of menopausal hot flushes: validation and cross-validation. Maturitas 25, 87–98 (1996). 8905599 10.1016/0378-5122(96)01046-8

[b21] Freedman R. R. & Blacker C. M. Estrogen raises the sweating threshold in postmenopausal women with hot flashes. Fertil. Steril. 77, 487–490 (2002). 11872200 10.1016/s0015-0282(01)03009-6

[b22] Freedman R. R. & Dinsay R. Clonidine raises the sweating threshold in symptomatic but not in asymptomatic postmenopausal women. Fertil. Steril. 74, 20–23 (2000). 10899491 10.1016/s0015-0282(00)00563-x

[b23] Carpenter J. S., Gilchrist J. M., Chen K., Gautam S. & Freedman R. R. Hot flashes, core body temperature, and metabolic parameters in breast cancer survivors. Menopause 11, 375–381 (2004). 15243274 10.1097/01.gme.0000113848.74835.1a

[b24] Stefanopoulou E. & Hunter M. S. Symptom perception in healthy menopausal women: Can we predict concordance between subjective and physiological measures of vasomotor symptoms? Am. J. Hum. Biol. 26, 389–394, 10.1002/ajhb.22530 (2014). 24590561

[b25] Bahr D. E. *et al.* Miniature ambulatory skin conductance monitor and algorithm for investigating hot flash events. Physiol. Meas. 35, 95–110, 10.1088/0967-3334/35/2/95 (2014). 24398586 PMC3951394

[b26] Freeman E. W. & Sherif K. Prevalence of hot flushes and night sweats around the world: a systematic review. Climacteric 10, 197–214, 10.1080/13697130601181486 (2007). 17487647

[b27] Rance N. E. & Young W. S. 3rd Hypertrophy and increased gene expression of neurons containing neurokinin-B and substance-P messenger ribonucleic acids in the hypothalami of postmenopausal women. Endocrinology 128, 2239–2247, 10.1210/endo-128-5-2239 (1991). 1708331

[b28] Nakamura K. & Morrison S. F. A thermosensory pathway mediating heat-defense responses. Proc. Natl. Acad. Sci. U. S. A. 107, 8848–8853, 10.1073/pnas.0913358107 (2010). 20421477 PMC2889337

[b29] Yoshida K., Li X., Cano G., Lazarus M. & Saper C. B. Parallel preoptic pathways for thermoregulation. J. Neurosci. 29, 11954–11964, 10.1523/JNEUROSCI.2643-09.2009 (2009). 19776281 PMC2782675

[b30] Dacks P. A., Krajewski S. J. & Rance N. E. Activation of neurokinin 3 receptors in the median preoptic nucleus decreases core temperature in the rat. Endocrinology 152, 4894–4905, 10.1210/en.2011-1492 (2011). 22028440 PMC3230049

[b31] Spooren W., Riemer C. & Meltzer H. Opinion: NK3 receptor antagonists: the next generation of antipsychotics? Nat Rev Drug Discov 4, 967–975, 10.1038/nrd1905 (2005). 16341062

[b32] Molnar G. W. Body temperatures during menopausal hot flashes. J. Appl. Physiol. 38, 499–503 (1975). 1150563 10.1152/jappl.1975.38.3.499

[b33] Meldrum D. R. *et al.* Elevations in skin temperature of the finger as an objective index of postmenopausal hot flashes: standardization of the technique. Am. J. Obstet. Gynecol. 135, 713–717 (1979). 495671 10.1016/0002-9378(79)90380-6

[b34] Meldrum D. R. *et al.* Gonadotropins, estrogens, and adrenal steroids during the menopausal hot flash. J. Clin. Endocrinol. Metab. 50, 685–689, 10.1210/jcem-50-4-685 (1980). 6444954

[b35] Casper R. F. & Yen S. S. Neuroendocrinology of menopausal flushes: an hypothesis of flush mechanism. Clin. Endocrinol. (Oxf.) 22, 293–312 (1985). 3884189 10.1111/j.1365-2265.1985.tb03243.x

[b36] Freedman R. R. Laboratory and ambulatory monitoring of menopausal hot flashes. Psychophysiology 26, 573–579 (1989). 2616704 10.1111/j.1469-8986.1989.tb00712.x

[b37] Otte J. L. *et al.* Comparison of subjective and objective hot flash measures over time among breast cancer survivors initiating aromatase inhibitor therapy. Menopause 16, 653–659, 10.1097/gme.0b013e3181a5d0d6 (2009). 19455068 PMC2817995

[b38] Sturdee D. W. & Reece B. L. Thermography of menopausal hot flushes. Maturitas 1, 201–205 (1979). 502877 10.1016/0378-5122(79)90009-4

[b39] Goodman R. L. *et al.* Kisspeptin neurons in the arcuate nucleus of the ewe express both dynorphin A and neurokinin B. Endocrinology 148, 5752–5760, 10.1210/en.2007-0961 (2007). 17823266

[b40] Topaloglu A. K. *et al.* TAC3 and TACR3 mutations in familial hypogonadotropic hypogonadism reveal a key role for Neurokinin B in the central control of reproduction. Nat. Genet. 41, 354–358, 10.1038/ng.306 (2009). 19079066 PMC4312696

[b41] Gianetti E. *et al.* TAC3/TACR3 mutations reveal preferential activation of gonadotropin-releasing hormone release by neurokinin B in neonatal life followed by reversal in adulthood. J. Clin. Endocrinol. Metab. 95, 2857–2867, 10.1210/jc.2009-2320 (2010). 20332248 PMC2902066

[b42] Lecci A. & Maggi C. A. Peripheral tachykinin receptors as potential therapeutic targets in visceral diseases. Expert Opin. Ther. Targets 7, 343–362, 10.1517/14728222.7.3.343 (2003). 12783571

