# Supplementary material for: Neurokinin B Administration Induces Hot Flushes in Women
Source: Sci Rep. 2015 Feb 16;5:8466. doi: 10.1038/srep08466 (PMC4329553; doi:10.1038/srep08466)
Supplement: Supplementary Information [file srep08466-s1.pdf]

Neurokinin B Administration Induces Hot Flushes in Women

Channa N. Jayasena[1#], Alexander N. Comninos[1#], Evgenia Stefanopoulou[2], Adam Buckley[1], Shakunthala Narayanaswamy[1], Chioma Izzi-Engbeaya[1], Ali Abbara[1], Risheka Ratnasabapathy[1], Julianne Mogford[1], Noel Ng[1], Zubair Sarang[1], Mohammad A. Ghatei[1], Stephen R. Bloom[1], Myra S. Hunter[2], Waljit S. Dhillo[1*]. [#]Joint first authors.

Supplementary Information Online

Supplemental methods

Neurokinin B

Human sequence neurokinin B was synthesised by Bachem (Merseyside, Liverpool, UK), and purified by reverse-phase high performance liquid chromatography (HPLC). Electrospray mass spectroscopy and amino acid analysis confirmed identity of the peptide (Lot:3007511). The Limulus amoebocyte lysate test detected no endotoxin (Associates of Cape Cod, Liverpool, UK), and bacterial culture was sterile (Department of Microbiology, Hammersmith Hospital, London, UK) in samples of neurokinin B peptide. Vials of freeze-dried neurokinin B were stored at -20°C and reconstituted in 0.9% saline.

Subjects

Five healthy female participants took part in study 1 (mean age 37.8±1.7y; mean body mass index 21.9±0.7kg/m^2) and ten healthy female participants took part in study 2 (mean age 35.3±1.3y; mean body mass index 22.1±0.7kg/m^2), following ethical approval (reference: 10/H0707/68) and written consent, in accordance with The Declaration of Helsinki. Participants had no medical problems, were not on any regular medication and had regular menstrual cycles. See Supplemental Tables 1 and 2 for participant characteristics for Study 1 and Study 2 respectively. Participants attended in the mornings and during the follicular phase of their menstrual cycle to minimise physiological changes in body temperature occurring during sleep and associated with ovulation (Day of menstrual cycle: Study 1, 8.0±0.9; Study 2, 5.9±0.6)[1].

Collection, processing and analysis of blood samples

Blood samples for serum analysis were collected in plain serum Vacutainer tubes (Beckton Dickson, Franklin Lakes, NJ, USA), and allowed to clot prior to centrifugation and separation. Serum LH, FSH and estradiol were measured using automated chemiluminescent immunoassays (Abbott Laboratories, Abbott Park, IL, USA). Reference ranges were as follows: LH (follicular), 2–10 IU/L; FSH (follicular), 4–14 IU/L; estradiol (follicular) <1000 pmol/L. The respective intraassay and interassay coefficients of variation were: 4.1 and 2.7% (LH); 4.1 and 3.0% (FSH); 3.6 and 3.4% (estradiol). Conversion factors for estradiol from International Units to Mass Units (pmol/L to pg/mL): x(1/3.67).

References:

1. Kelly G. Body temperature variability: A review of the history of body temperature and its variability due to site selection, biological rhythms, fitness, and aging. *Altern Med Rev* **11(4),** 278-293 (2006).

Supplementary Figure S1

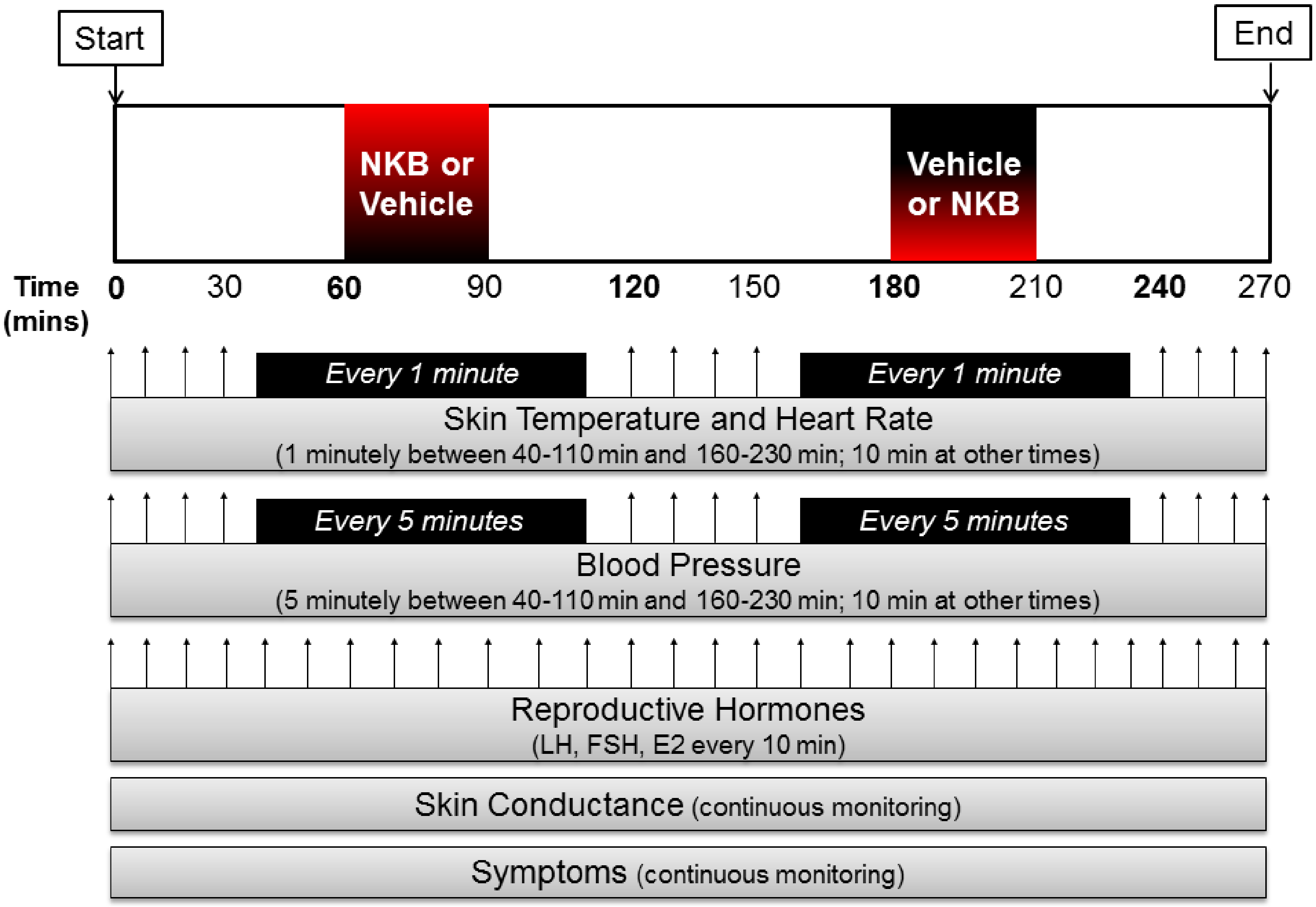

Supplementary Figure S1 online. Protocol diagram for double-blinded administration of vehicle and neurokinin B (Study 2). Ten healthy women were admitted for a 270 minute study during days 2-10 of their menstrual cycle to a climate-controlled Clinical Research Unit with ambient temperature 24oC and relative humidity 50%. Each participant received one 30 minute neurokinin B infusion (NKB, 5.12nmol/kg/h) and one 30 minute vehicle infusion, in random double-blinded order. Skin temperature and heart rate were recorded every minute for 20 minutes pre-, during, and 20 minutes post-infusions and every 10 minutes at all other times. Blood pressure was recorded every 5 minutes from 20 minutes pre-, during, and 20 minutes post-infusions and every 10 minutes at all other times. Reproductive hormones: luteinizing hormone (LH), follicle-stimulating hormone (FSH) and estradiol were measured every 10 minutes. Skin conductance and symptoms were assessed continuously.

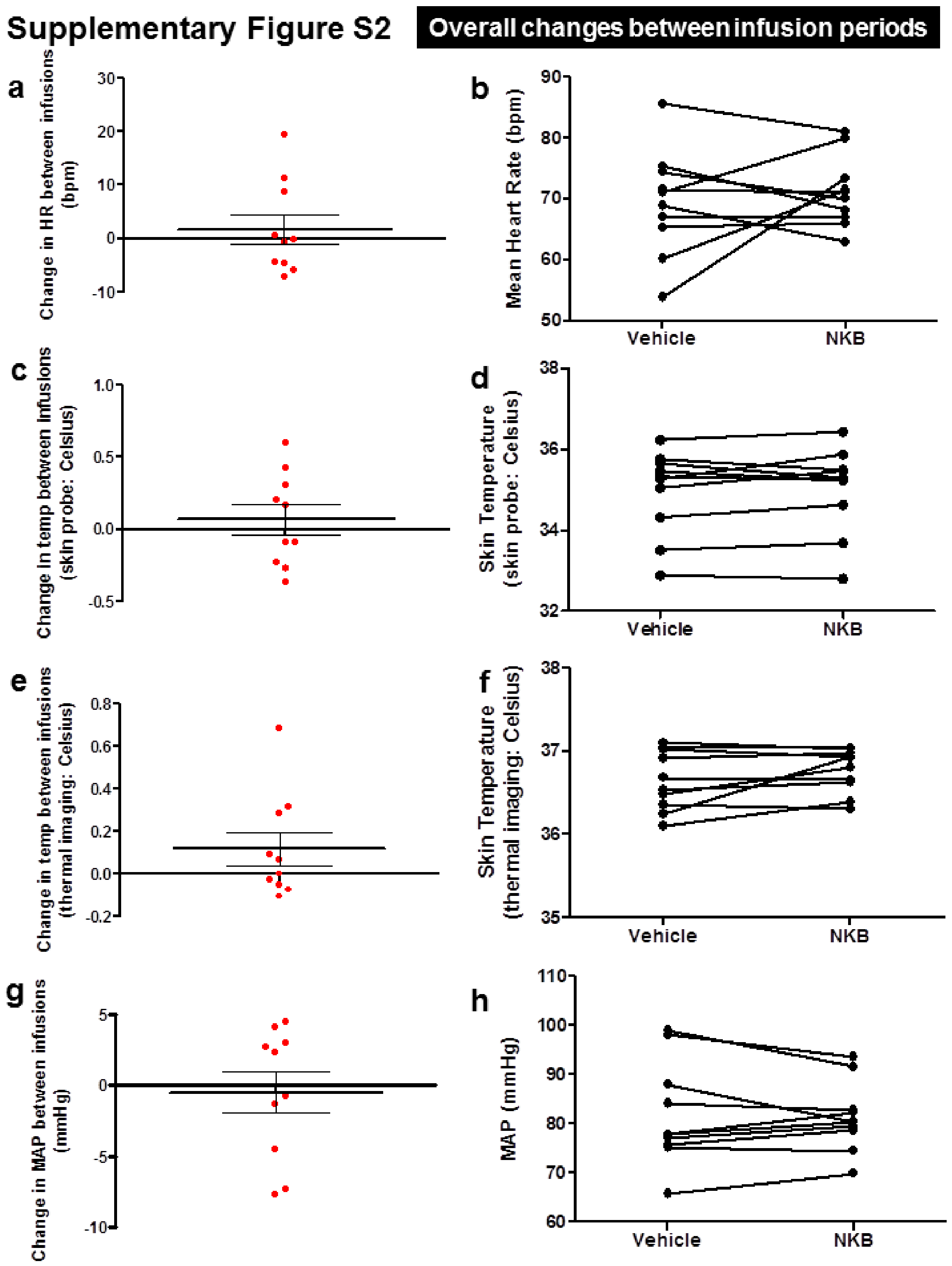

Supplementary Figure S2 online. Overall changes in objective physiological parameters during neurokinin B (NKB) and vehicle infusions. In order to determine if NKB administration *per se* altered heart rate (**a**), skin temperature by skin probe (**c**), skin temperature by thermal imaging (**e**) and mean arterial pressure (MAP) (**g**), mean values for each parameter were compared between the entire 30 minute infusion periods of neurokinin B (NKB) and vehicle. Parts **b**, **d**, **f** and **h** represent absolute changes in each physiological parameter. Data presented as mean±SEM.

Supplementary Table S1 online: Participant Characteristics for Study 1. Age, follicular day of menstrual cycle, body mass index (BMI), and baseline reproductive hormone levels at start of study visit.

**Participant**	Age (years)	Day of menstrual cycle	BMI (kg/m^2)	Baseline LH (IU/L)	Baseline FSH (IU/L)	Baseline Estradiol (pmol/L)
**1**	42	5	22.2	5.10	7.16	67
**2**	32	10	20.8	3.42	3.67	756
**3**	40	9	24.4	3.41	4.19	278
**4**	39	9	20.4	5.16	6.64	388
**5**	36	7	21.6	4.33	3.58	291
**mean±SEM**	37.8±1.7	8.0±0.9	21.9±0.7	4.3±0.4	5.0±0.8	356±113

Supplementary Table S2 online: Participant Characteristics for Study 2. Age, follicular day of menstrual cycle, body mass index (BMI), and baseline reproductive hormone at start of study visit.

Participant	Age (years)	Day of menstrual cycle	BMI (kg/m^2)	Baseline LH (IU/L)	Baseline FSH (IU/L)	Baseline Estradiol (pmol/L)
**1**	40	6	24.4	2.06	4.60	207
**2**	32	10	24.2	2.87	2.87	947
**3**	39	5	20.4	4.31	3.61	421
**4**	37	6	18.6	4.53	6.51	249
**5**	32	4	20.8	2.15	6.97	189
**6**	36	3	24.9	3.31	4.13	93
**7**	26	6	22.7	5.42	4.85	173
**8**	36	5	22.9	4.38	4.29	98
**9**	36	6	22.0	2.79	3.51	707
**10**	39	8	20.2	5.20	3.83	984
**mean±SEM**	35.3±1.3	5.9±0.6	22.1±0.7	3.7±0.4	4.5±0.4	407±109
